# Technologies for Clinical Diagnosis Using Expired Human Breath Analysis

**DOI:** 10.3390/diagnostics5010027

**Published:** 2015-02-02

**Authors:** Thalakkotur Lazar Mathew, Prabhahari Pownraj, Sukhananazerin Abdulla, Biji Pullithadathil

**Affiliations:** Nanosensor Laboratory, PSG Institute of Advanced Studies, Coimbatore641 004, India; E-Mails: tlazarmathew@gmail.com (T.L.M); prabhaharip@gmail.com (P.P); sukhananazerin@gmail.com (S.N.A); bijipsgias@gmail.com (B.P.)

**Keywords:** breath analysis, noninvasive techniques, biomarkers, metabolism, nanosensors

## Abstract

This review elucidates the technologies in the field of exhaled breath analysis. Exhaled breath gas analysis offers an inexpensive, noninvasive and rapid method for detecting a large number of compounds under various conditions for health and disease states. There are various techniques to analyze some exhaled breath gases, including spectrometry, gas chromatography and spectroscopy. This review places emphasis on some of the critical biomarkers present in exhaled human breath, and its related effects. Additionally, various medical monitoring techniques used for breath analysis have been discussed. It also includes the current scenario of breath analysis with nanotechnology-oriented techniques.

## 1. Introduction

Medical monitoring technologies and diagnostics are the essential tools that assist clinicians in discriminating health from disease states and have the potential to envisage impending effects [1]. The detection of diseases strengthens the possibility for successful treatment and also insists the demand for cheap, noninvasive, and qualitative diagnosis of diseases. The first noninvasive method validated to assess airway inflammation was the examination of induced sputum. Sputum analysis could not set up itself as a routine method since it is time-consuming, convoluted and expensive. Therefore, researchers seek out the biomarkers directly in the expired air [2]. The metabolite that has been measured in media other than blood has significantly increased because of the major demands for noninvasive diagnostics. Breath is recognized as a medium that can be measured through doses of chemicals from clinical and environmental conditions [3]. Ancient Greek physicians found that the aroma of human breath provides clues to some diseases and supplies an insight into physiological and pathophysiological processes in the body [4].

### 1.1. Human Exhaled Breath Gas Analysis

**Figure 1:**
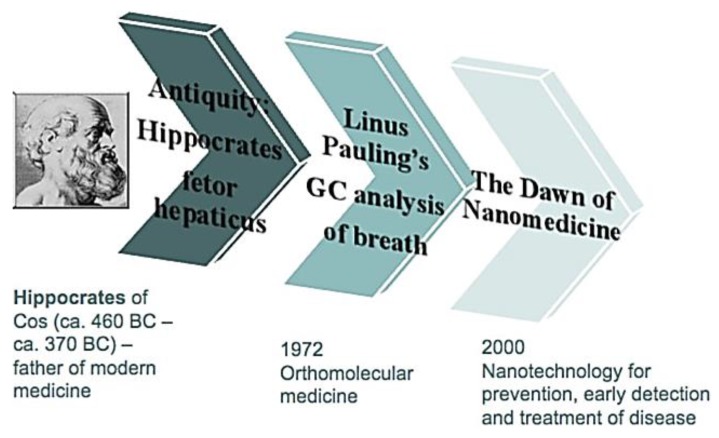
Timeline of expired breath gas analysis, Reproduced with permission from [8]. Copyright 2011, SPIE.

Breath gas analysis is a promising scientific field with a large scientific community globally spread with a major impact on many application domains. It is the noninvasive diagnostic method for a holistic measure of patient’s physiological status. It elucidates the physiological basis of exchange of gases between air and blood. It has potentially great advantages compared to other diagnostics including blood, urine, biopsy, endoscopy, imaging as it is completely noninvasive and implies virtually unlimited repeatability with respect to frequency, access and cost [5]. Breath samples are somewhat easier to obtain than serum or urine samples and advantageous within a myriad of sample collections [3]. It is an inexpensive method with fast turnaround time and minimal biohazard waste. The breath of patients has been characterized by a specific odor since the time of Hippocrates and it began with the discoveries of Lavoisier in the late 1780s. He identified CO_2_ as a major constituent in the exhaled breath. In 1971, Linus Pauling identified a large number of volatiles in human breath. He breathed through a very cold tube and analyzed the frozen compounds by gas chromatography and found that human breath contains a large number of volatile organic compounds (VOCs) in picomolar to nanomolar concentrations [6]. The main advantage of the expired gas analysis is that it can be used on people of all ages and conditions and it poses no risk to the patients [7]. Figure 1 shows the timeline of expired breath gas analysis.

Haick *et al*. [9] discussed the biochemical origin of VOCs related to the cancer and the relation between the VOCs in the exhaled breath as well as in the blood. They discussed that the VOCs in the breath have more advantages than those in the blood. It was reported that comprehensive work has yet to be carried out for breath analysis, but nanomaterials based sensors can be used as highly selective sensors as it could provide high sensitivity. The breath analysis should eventually fit into a portable device. They presented a list of 115 cancer related VOCs published in the literature. Amann *et al*. [10] reported the assessment of exhalation kinetics of VOCs related to the cancer. These 112 different compounds have diverse storage compartments in the body and their exhalation kinetics relies on one or more combination of the factors such as the concentrations of VOCs in various parts of the body, the VOCs production and metabolism rates, the partition coefficients between tissue(s), blood and air and the VOCs diffusion constants. They emphasized that 112 VOCs in the exhaled breath are linked to the potential markers of cancer. Broza *et al*. [11] presented the nanomaterial based sensors for the detection of VOCS. This review focused on optical and mechanical transducer which incorporates the important classes of nanomaterials and the integration into selective and cross selective sensor arrays were also discussed. The integration possibilities towards different types of nanomaterials into sensor arrays and the expected outcomes and limitations were also briefly elucidated. Hakim *et al*. [12] reported the VOCs which are responsible for lung cancer. The biochemical pathways of the lung cancer related VOCs such as hydrocarbons, alcohols, aldehydes, ketones, esters, nitriles, and aromatic compounds were discussed. In this review, we mainly focused on major exhaled breath gases, its metabolism and related diseases. Additionally, various techniques for the breath analysis, and its advantages were also emphasized. Nanomaterial-based sensor for breath analysis should be sensitive at very low concentrations of volatile organic compounds. A sensing matrix based on nanomaterials becomes a clinical and laboratory diagnostic tool because they are considerably smaller in size, easier-to-use, and inexpensive compared tospectrometry or spectroscopy. It gives rapid response and proportionately to small changes in concentration and provides a consistent output that is specific to a given volatile organic compound. When there is no contact with the VOCs, the sensor must rapidly come back to its baseline state or be simple and inexpensive enough to be disposable. Several reviews have focused on the biochemical, methodological and clinical aspects of breath analysis in attempts to bring breath testing closer to practice for comprehensive disease detection. It gives some specific attention to the technological gaps and perplexing factors that impede nanomaterial-sensor-based breath analysis and it enthralls the future research and development efforts towards the possible approaches to overcome these obstructions. It has been used for clinical diagnosis, environmental conditions and, moreover, some of the applications rely on technical developments and clinical evaluations [13].

### 1.2. Human Breath

**Figure 2:**
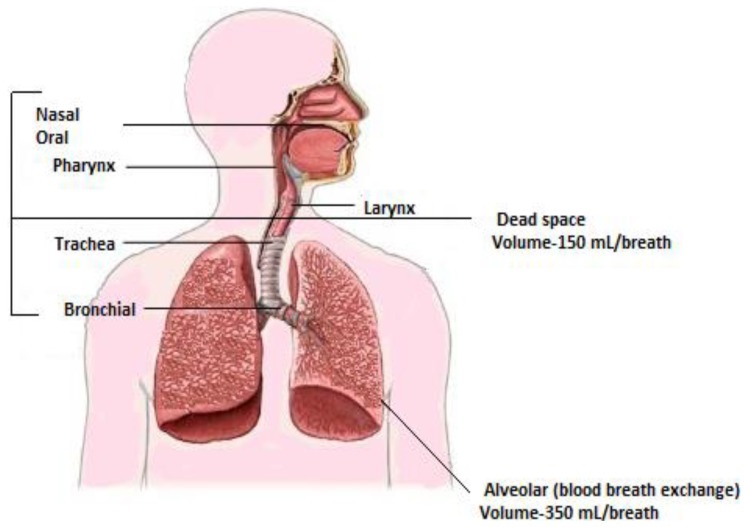
Respiratory system and its relation to breath. Adapted with permission from [15], copyright 2011, American Chemical Society.

Breath can be classified as inhaled breath and exhaled breath. Inhalation allows the external environment to exchange with the internal environment at the blood air barrier within the alveoli of the lungs. Exogenous compounds diffuse into blood and allow them to make contact with virtually every tissue in the body. During exhalation, endogenous compounds reflecting internal bodily conditions diffuse from the blood into the breath [13]. A healthy person exhales half a liter or more in expiration, and the first 150 mL of expiration consists of dead space air from the upper airways, where no gas exchange has occurred as depicted in Figure 2. Gas exchange occurs at the surface of several petite chambers known as *alveoli* at the tip of bronchial air passage. The first half of expiration is removed to reduce the dilution and contamination of alveolar air by dead space air [3,14].

In exhaled breath, more than 3500 different components have been found and the list is continually growing. The complex matrix of exhaled breath component is termed as molecular breath signature [2]. The actual breath is a bulk matrix and contains mixtures of nitrogen, oxygen, carbon dioxide, inert gases, water vapor and a contain thousands of trace volatile organic compounds (VOCs) and inorganic molecules such as NO, NH_3_ or CO. VOCs commonly found in normal breath are acetone, ethane and isoprene which are nothing but the metabolic products of expired breath constituents and their concentrations range from parts per million (ppm) to parts per trillion (ppt). The composition of breath varies from each individual person both quantitatively and qualitatively [16]. Table 1 illustrates the major components and its concentrations in the exhaled breath for healthy persons. Elevated levels of a number of these compounds are indicative of systemic disorders and extra pulmonary organ failures. These are termed as systemic biomarkers. Breath analysis has impelled the exploration of airway diseases by estimation of biomarkers deriving from the airways and lung structures. These can be referred to as lung biomarkers. Human breath contains a myriad of biomarkers resulting from the blood by passive diffusion across the pulmonary alveolar membrane [2]. Breath testing offers a novel approach to the disease diagnosis, evaluation of various common disorders, and assessment of exposure to VOCs [17].

**Table 1:** Major compounds and its concentrations for healthy persons.

Exhaled Breath	Concentration	Reference
Nitrogen	78.04%	[18]
Oxygen	16%	[18]
Carbon dioxide	4%–5%	[18]
Hydrogen	5%	[18]
Carbon monoxide	0–6 ppm	[19]
Ammonia	0.5 ppm–2 ppm	[20]
Inert gases and VOCs: Acetone, Isoprene and Ethanol	0.9% <1 ppm	[21]
Hydrogen sulphide	0–1.3 ppm	[22]
Nitric oxide	10 ppb–50 ppb	[6]
Nitrous oxide	1 ppb–20 ppb	[6]
Carbonyl Sulphide	0–10 ppb	[6]
Ethane	0–10 ppb	[6]
Pentane	0–10 ppb	[6]
Methane	2 ppm–10 ppm	[6]
Relative Humidity:		
1. Oral Exhalation	91%–96%	[18]
2. Nasal Exhalation	82%–85%	[18]
Temperature Range	Between 34 °C and 36 °C	[18]

## 2. Interfering Expired Gases and Its Effects

Elevation of breath levels of some of these gases (ammonia, ethane, isoprene, butane, nitric oxide, ethanol, carbon monoxide, methane, hydrogen sulphide, and acetone) is associated with a variety of metabolic and pathologic conditions [23]. Breath VOCs are the most prevalent components within the wide range of constituents that can be detected. These can be considered as volatile biomarkers within the exhaled breath matrix. The most concentrated endogenous constituents in breath are isoprene (12–580 ppb), ethanol (13–1000 ppb), methanol (160–2000 ppb), and acetone (1.2–1800 ppb); other alcohols are present in the very low ppb concentration range [24].

### 2.1. Ammonia Metabolism

**Figure 3:**
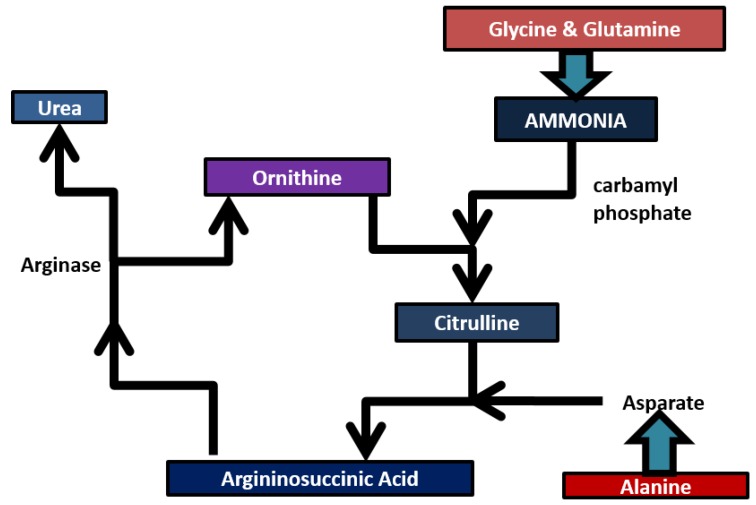
Ammonia and its metabolites.

Ammonia (NH_3_) plays an important role in the human body. It is considered to be an important biomarker. Ammonia is present in all body fluids as ammonium ion and also in the form of NH_3_. It becomes toxic to the human body when the concentration is higher. Ammonia is present as gaseous NH_3_ in the air around the alveolar interface in the lungs due to its volatile nature. Blood ammonia is tightly regulated via the urea cycle for healthy persons and the concentration of ammonia represents more requirements in medicine since it is a molecule produced during protein metabolism and involved in numerous health and disease states [21]. A fine balance of nutritional absorption and toxin removal takes place when food is ingested. The stomach, lumen and intestines break down food into nucleotide bases, amino acids and other nitrogenous compounds which diffuse into the blood. These nitrogenous compounds are absorbed from the blood into the liver which converts them into less toxic soluble forms which can be safely removed in relatively low volumes of water [24,25]. Figure 3 represents the metabolism of ammonia in humans. Ammonia is absorbed into the liver and combined with carbon dioxide to form carbamoyl phosphate. It enters into the urea cycle and reacts with ornithine to form citrulline. Amino acids are directed into the urea cycle via their transamination by aspartate, which combines with citrulline to form argininosuccinate [26].

Aspartate acts to utilize the availability of free ammonia used in the initial steps of urea cycle with carbon dioxide. Argininosuccinate is then divided into fumarate and arginin. Then arginine reacts with arginase and water to produce urea and regenerated ornithine [25]. As the liver finishes the process, the urea is excreted in the bloodstream along with the excess ammonia and is absorbed by the kidneys via the glomerulus [27]. The ammonium ion resulting from deamination of amino acid (glutamate) is converted to ammonia either directly or indirectly. Glutamine and alanine are the carriers of ammonia (from muscle and other tissues to the liver). Kidneys serve the purpose of filtering the blood urea and excess ammonia out of the body in the form of urine [26]. The liver and kidneys play a significant role in removing the ammonia from the body. If there is a problem associated with the proper removal of ammonia from the kidneys and liver, ammonia levels in the blood may tend to increase.

Kidney failure is one of the diseases identified by extremely high ammonia content in human expired breath gas. The ammonia odor in the mouth of kidney failure patients is associated with high levels of blood urea nitrogen (BUN). When the kidneys are not functioning well, BUN accumulates in the human body. The excess of urea will be decomposed into ammonia under the effect of urea enzymes in the gastrointestinal tract. High BUN in saliva is decomposed into ammonia to cause the smell of ammonia in the mouth [28]. Ammonia levels in the blood rise when the liver is unable to convert ammonia to urea which leads to cirrhosis or severe hepatitis. With liver dysfunction, the result is hyperammonaemia and the further consequences including damage to brain tissue (hepatic encephalopathy). Elevated levels of ammonia are the most responsible for hepatic encephalopathy. The brain is protected by a blood-brain barrier that prevents toxic substances entering into the body. However, if there is a barrier in the synthesis of the urea cycle, components can modify the permeability of the blood-brain barrier. For instance, a compound that can do this is glutamine. Glutamate can react with excess ammonia via glutamine synthetase to create glutamine during the transamination process in the urea cycle. Elevated levels of glutamine are then able to change the osmotic tendencies around brain tissue resulting in swelling of the brain [25]. Swelling is due to the higher concentrations of toxins outside the barrier flowing into the lower concentrated area of the brain. Ammonia is capable of modifying the gene expression and signal transmission of astrocytes and neurons when entering into the brain. These modifications primarily induce type II Alzheimer’s disease. Even though glutamine production can cause damage to the brain, it prevents cell damage. Astrocytes generate glutamine synthetase which catalyzes the reaction of ammonia with glutamate to reduce the ammonia levels [29]. Breath ammonia is used to diagnose peptic ulcers affecting either the stomach or duodenum. The link between these ulcers and breath ammonia is a bacterium known as *Helicobacter pylori*. *H. pylori* allows biological acids to deteriorate the tissue and form ulcers [30]. If *H. pylori* is present in the stomach the high levels of excreted urease are detected by monitoring the breakdown of the labeled urea into radioactive carbon dioxide and ammonia [31]. Initial ammonia baseline levels can vary from individual to individual, the rate of ammonia increase upon the absorption of urea. However, there are bacteria which can generate ammonia in the oral cavity [32]. In the mouth, anaerobic bacteria metabolize food debris and create numerous byproducts which are the cause of the smells associated with halitosis. Ammonia in the oral cavity has the potential for assessing halitosis and oral hygiene. Halitosis is primarily due to volatile sulphur compounds (VSCs) in the oral cavity causing tissue damage and malodour [33]. There is a 0.39 correlation between VSCs and ammonia [34]. Lung dysfunction or impairment such as asthma has potential links with breath ammonia. The individuals with asthma have lower levels of ammonia in their breath than healthy individuals and also the concentrations of ammonia produced by glutaminase may be directly affected by the levels of corticosteroids and cytokines produced by asthma patients [35]. Thus excess concentration of ammonia in expired air can be utilized to predict possibility of the detection of diseases such as kidney failure, cirrhosis or hepatitis, hepatic encephalopathy, peptic ulcers, halitosis and asthma. Efforts are being made to develop devices to detect this biomarker for expired breath air analysis.

### 2.2. Acetone Metabolism

**Figure 4:**
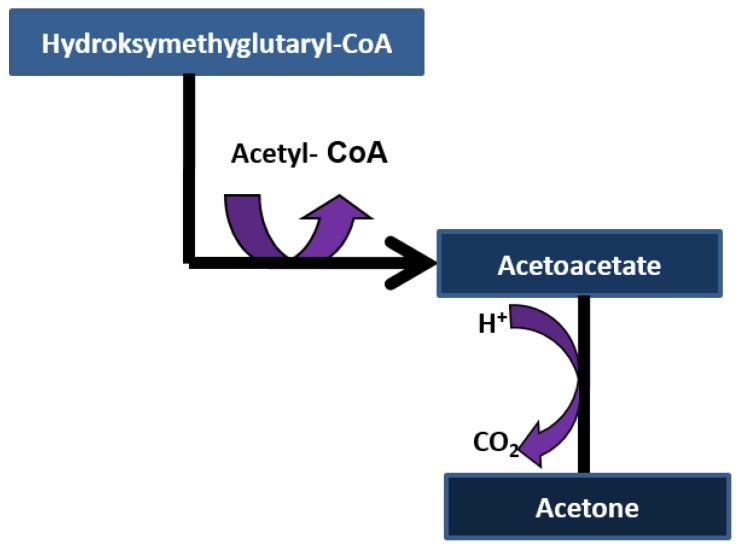
Decarboxylation of ketone bodies.

Acetone ((CH_3_)_2_CO) is the volatile organic compound and it is colorless. The sweet “aroma of decaying apples” for patients with severe diabetes was first identified by John Rollo in 1798. In 1857, Petters found that the sweet aroma has its origin from acetone [36,37]. Acetone can be excreted in breath and urine and undergoes *in vivo* metabolism [38]. Acetone is a metabolite and produced by lipolysis, absorbed into the blood stream, and expelled through alveoli of the lungs during exhalation [39]. Acetone is a three-carbon ketone body derived from oxidation of non-esterified fatty acids and resulting from acetoacetate through spontaneous decarboxylation or enzymatic conversion (via acetoacetate decarboxylase) as shown in Figure 4 [21]. Acetol (1-hydroxyacetone) and 1,2-propanediol (PPD) are the two possible metabolites of acetone [38]. For long time it has been known that breath acetone is correlated with ketone bodies in plasma, blood acetone and β-hydroxybutyrate in plasma. The relationship between blood and breath acetone is linear (acetone in exhaled air is approximately 1/330 of the acetone in plasma) [36].

Acetone is normally produced by humans in baseline conditions with very little circadian fluctuations [18]. Since elevated levels of acetone is excreted in exhaled air as a consequence of the ketosis, it has been anticipated to measure human breath acetone to predict and detect diabetic ketoacidosis, instead of blood- and urine sampling [36]. Breath acetone levels ranges from a relatively high 0.5 ppmv for healthy persons to hundreds of ppmv for critically ill, ketoacidotic diabetics [40]. Breath acetone could be used as an auxiliary tool for the diagnosis and early screening of diabetes [38]. If the blood glucose level is quite high for an extended period of time, fatty acids and amino acids are burned and produce energy. In this case, slag products such as ketone bodies are produced. The ketone bodies are stored in the blood, which lowers the Ph concentration. Glucose is not available as a source of energy for the persons with untreated diabetes. Consequently, ketone bodies (acetone, acetoacetate and β-hydroxybutyrate) are produced as by-products and energy source when fat is broken down instead of glucose. High levels of ketones are produced as a result of low insulin levels for diabetic ketoacidosis [36]. Elevated acetone production is also a normal metabolic response in healthy persons due to physical exercise [40]. Breath acetone concentration typically varies from 0.2 to 2.4 ppm in fasting healthy adults. Breath acetone increases during diabetic ketoacidosis, fasting, and high-fat diets [41].

### 2.3. Isoprene Metabolism

**Figure 5:**
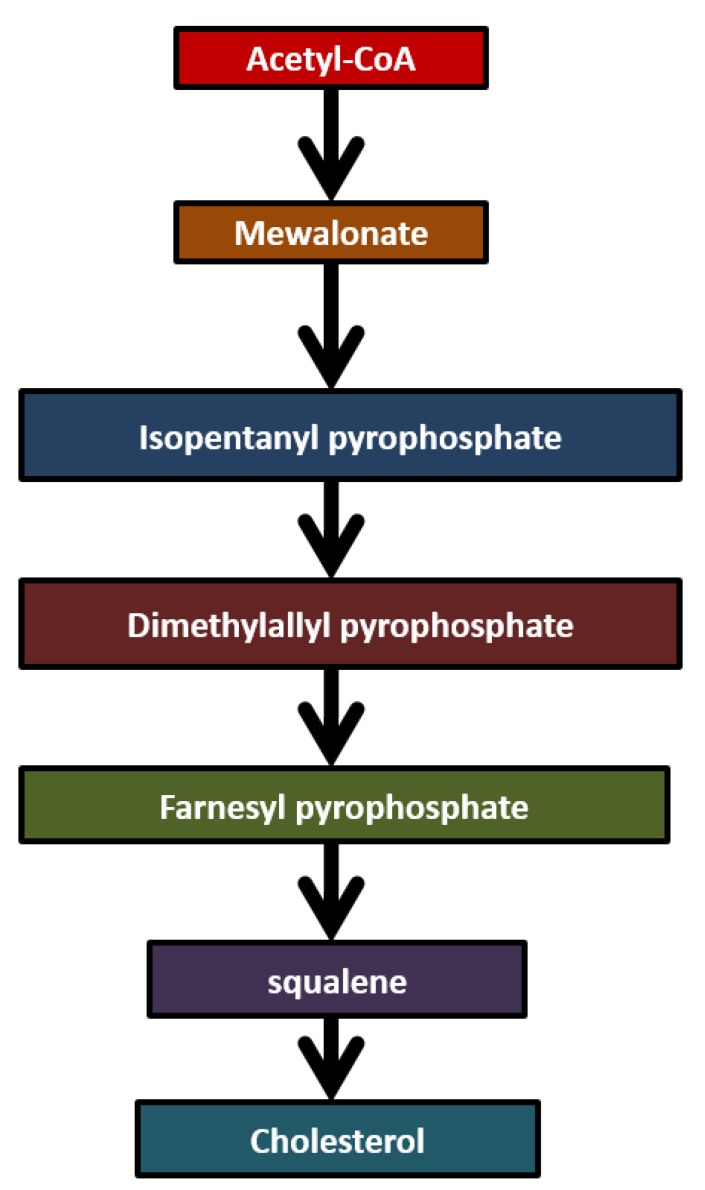
Biochemical pathway of isoprene.

Isoprene (2-methyl-1,3-butadiene) is an unsaturated hydrocarbon. Isoprene is one of the most abundant hydrocarbons in human breath. Isoprene is the most abundant biogenic hydrocarbon emitted by the earth’s vegetation and it is also the major hydrocarbon that is endogenously produced by mammals. The primary source of isoprene in the human body has been ascribed to the mevalonate pathway of cholesterol biosynthesis as shown in Figure 5. It is originating from acetyl-CoA and mevalonate is transformed into dimethylallyl pyrophosphate (DMPP). Metabolization of isoprene in mammals primarily rests on epoxidation by cytochrome [42]. Isoprene is highly abundant in human breath and accounts for up to 70% of total hydrocarbon removal via exhalation. Isoprene is considered as a by-product of cholesterol synthesis. It appears to be a useful metabolic biomarker for several metabolic disorders. The concentration of isoprene in breath has been widely studied in human subjects undergoing general anesthesia [18]. Isoprene increases with age and is independent of metabolic state in diabetic children. Mendis *et al*. [43] demonstrated an increase in breath isoprene in patients experiencing acute myocardial infarction. The concentration of isoprene in the breath of heart failure patients was significantly lower than that found in controls. Breath isoprene may serve as a sensitive and non-invasive indicator for assaying several metabolic effects in the human body. However, isoprene concentrations in exhaled human breath exhibit a large variability and therefore it may be difficult to make devices using this biomarker. In children and adolescents, isoprene excretion in breath appears to increase with age. Being a by-product of cholesterol biosynthesis, breath isoprene has been as an additional diagnostic parameter of patients suffering from lipid metabolism disorders such as hypercholesterolemia [44]. Isoprene, acetone and methanol are compounds commonly present in exhaled breath. These three compounds from the exhaled breath show slightly lower concentrations in lung cancer patients as compared to healthy patients. The median concentration of isoprene in exhaled breath of cancer patients is 81.5 ppb, whereas in healthy controls the concentration of isoprene is 105.2 ppb [45].

### 2.4. Methane Metabolism

Methane (CH_4_) producing bacteria are capable of producing methane only in the presence of hydrogen. Methane is produced by methanogens such as *Methanobrevibacter smithii* (obtain from hydrogen or carbon dioxide) and *Methanosphaera stadtmanae* (obtain from methanol and hydrogen in the intestine) [46]. Methane is considered to be majorly produced in the gut by hydrogenation of carbon dioxide by methanogens. Other microorganisms in the human gut are also capable of producing methane, such as certain Clostridium and Bacteroides species. Methane is not produced in the breath until the methanogens reach a density of about 108 methanogenic bacteria·g^−1^ stool. The methanogens have a controlled metabolism in which they must reduce simple substrates to methane in order to produce cellular energy [47]. It is absorbed from the intestinal mucosa, dissolves in the blood and reaches the lungs, where it is subjected to gas exchange and is then expelled in the breath [46]. Bacterial CH_4_ present in the intestinal tract is excreted via the lungs, and breath testing has therefore become a tool with which to diagnose certain gastrointestinal conditions [48]. It has been reported that *Methanobrevibacter* have been identified in the feces in case of high concentration of methane in the breath, whereas there is only a low frequency of detection of this type of bacteria in the case of low concentration of methane. The concentration of methane ejected in the breath varies in connection with illnesses such as colorectal cancer and inflammatory bowel disease. Breath methane tests and culture based methods have traditionally been used to characterize methanogen populations [46]. Methane is released during stress conditions, and it has been suggested that the excretion of methane in the breath of living things may reflect intestinal bacterial fermentation [48].

### 2.5. Hydrogen Sulphide Metabolism

H_2_S was primarily viewed as a toxic gas and as an environmental hazard. H_2_S is identified as a gaseous biological mediator that is produced in mammalian species, including humans. H_2_S is synthesized endogenously by two pyridoxal-5′-phosphate-dependent enzymes responsible for metabolism of l-cysteine, cystathionine beta-synthase (CBS) and cystathionine gamma-lyase (CSE) [49]. H_2_S is the third endogenous gasotransmitter to be discovered after nitric oxide and carbon monoxide. Production of H_2_S in the human brain has been attributed to CBS whereas CSE has been detected in vascular smooth muscle and endothelial cells. Production of H_2_S in vascular endothelial cells is triggered by muscarinic cholinergic activation [48]. H_2_S stands for a promising biomarker for asthma. Serum H_2_S correlated with the severity of different respiratory diseases and airway inflammation. It appears that serum H_2_S may be used as a marker for airway inflammation and lower respiratory tract infections [50,51]. However, serum H_2_S level can be affected by many non-respiratory diseases. To detect blood H_2_S level is both non-specific and invasive. Interestingly, H_2_S shares a lot of attributes with NO and also exist in breath, which means it can be sampled noninvasively [16]. Detecting H_2_S levels in breath is the viable method for the measurement of systemic and/or airway levels. The detection of H_2_S in exhaled breath is attractive because it represents better physiological conditions due to minimal moisture loss and contamination by oral sources. Besides containing very low concentrations of H_2_S, breath samples are characterized by high moisture levels [52]. For a long time, it has been known that intravenous injection of a solution of H_2_S gas results in its exhalation within seconds [53]. H_2_S is originating from the lower respiratory tract in humans [54]. H_2_S present in the breath was around 1.5 ppb, compared with 1.2 ppb in the ambient air. Oral bacteria produce H_2_S, and this can be detected in the breath. The potential of breath H_2_S measurement is being used as a biomarker of airway inflammation [55,56]. H_2_S appears to be a mediator of key biological functions including life span and survivability under severely hypoxic conditions [57,58]. Hydrogen sulphide, as a vascular relaxant agent, may be a participant in the regulation of cardiovascular function. Hydrogen sulfide and other volatile sulfur compounds identified in the breath are usually associated with oral malodour [59]. H_2_S in exhaled breath would not accurately reflect H_2_S metabolism in the respiratory system because it will be affected by pathophysiological conditions of oral and dental health. Bacteria-originated H_2_S would also affect H_2_S level in exhaled breath. Exhaled nasal H_2_S may particularly reflect the healthy or disease status of the lung and airway tissues. The technological and instrumental challenges for detecting H_2_S in exhaled air would assist to expose the potential of H_2_S as a biomarker for respiratory-specific diseases, such as asthma [49].

### 2.6. Nitric Oxide Metabolism

Nitric Oxide (NO) is a reactive free radical as well as diffusible gas [60]. The detection of NO from the human expired breath was first reported by Gustafsson *et al*. [61]. It is a by-product of the oxidation of l-arginine to l-citrulline via three isoforms of synthase. These are constitutively expressed nitric oxide synthase I (NOS1), NOS3, and inducibly expressed NOS2 [12]. NO is a biomarker of respiratory disease such as asthma and it is normally produced by human respiratory tract mucosa. NO is unstable in biological tissues [62] and difficult to perform as gas reacts quickly with hemoglobin or other Fe^2+^ containing proteins. NO in a gas phase is fairly stable at low concentrations [63]. The elevated levels of NO in asthmatic patients are highly imputed to activate the NOS2 by damaging airway epithelial cells and by inflammation. It also partially activates the NOS1 [64,65]. The NO concentration below 25 ppb is normal, and above 50 ppb is prone to cause airway inflammation [66]. The concentration of NO is high and mainly found in the human breath for inflammatory respiratory disorders including sinus disease, viral upper respiratory tract infection and lung transplant rejection [67,68]. Nevertheless, the levels of NO are variable in patients with chronic obstructive pulmonary disease (COPD) and cystic fibrosis [69,70].

### 2.7. Ethane and Pentane Metabolism

Hydrocarbon gases, produced by the oxidation of cellular lipids, such as pentane and ethane [71]. Hydrocarbons can be produced by a free radical chain reaction mechanism during lipid peroxidation of polyunsaturated fatty acids (PUFAs) of cell membranes, and also be produced in a lesser amount by protein oxidation and colonic bacterial metabolism. Ethane and pentane can be produced by the decomposition of ω-3 and ω-6 polyunsaturated fatty acids. Pentane accumulates in human fat depots and gradually released over a period of several days whereas ethane can be expired in the human breath within minutes of their formation as it has low solubility in the blood and tissues [72]. Pentane and ethane levels can be increased in patients with asthma [73,74], COPD [75], obstructive sleep apnea [76], and ARDS [77,78]. Spittle *et al*. [79] found that the concentration of ethane increased during hemodialysis. Exhaled ethane is a biomarker for oxidative stress [80]. Pentane and ethane levels are also increased by both physical [81,82] and mental [83] stress. Pentane is high in the human breath for the patients with sepsis or SIRS [78]. Pentane can be detected in the human breath and it causes oxidative stress, inflammatory bowel disease [84], sleep apnea [85] cancer [86] and ischemic heart disease [87]. Table 2 shows the biomarkers and its sources for various diseases can be analyzed from the human breath.

**Table 2:** Biomarkers, sources and diseases.

Biomarkers	Sources	Diseases
Ammonia	Protein Metabolism	kidney failure, cirrhosis or hepatitis, hepatic encephalopathy, peptic ulcers, halitosis and asthma
Acetone	Acetoacetate Decarboxylation	Diabetes, lung cancer, dietary fat losses, congestive heart failure, brain seizure
Isoprene	Mevalonate Metabolism	disorders in cholesterol metabolism such as hypercholesterolemia
Methane	Intestinal bacteria metabolism of carbohydrates	Intestinal problems, colonic fermentation
Hydrogen Sulphide	metabolism of l-cysteine, cystathionine beta-synthase (CBS)	airway inflammation
Nitric Oxide	Nitric oxide Synthase	Asthma, acute lung injury, ARDS, inflammatory lung diseases, lung infection, lung cancer, rhinitis
Ethane	Peroxidation of polyunsaturated fatty acids	Oxidative stress, vitamin E deficiency, breast cancer, active ulcerative colitis
Pentane	Peroxidation of polyunsaturated fatty acids	Liver diseases, schizophrenia, breast cancer, rheumatoid arthritis, oxidative stress, acute myocardial infarction, asthma

## 3. Techniques for Breath Gas Analysis

There are various techniques by which expired breath gases can be analyzed. Exhaled breath can be captured by various methods and analyzed for a wide range of biomarkers from metabolic end products to proteins [88]. A successful diagnostic technique should be selectively identified a unique species in such a complex matrix [3]. Some of the techniques can be summed up as follows.

### 3.1. Gas Chromatography (GC)

Gas Chromatography has been used in the analysis of trace level compounds in the exhaled breath. In this technique, samples are injected into the headspace of the chromatographic column. It is separated by gaseous mobile phase and separation efficiency relies on the GC column. The non-polar compound (silicone) of this column is separated on the basis of the boiling point and the polar compound of this column is separated on the basis of polarity of the substances [89]. There are a few types of detection methods employed with GC for substance which is present in the breath [14]. These are Mass Spectroscopy (MS), Flame Ionized Detection (GC-FID), Ion Mobility Spectroscopy (IMS), Electrolyzer Powdered Flame Ionization (EFID).

#### 3.1.1. Gas Chromatography-Mass Spectrometry (GC-MS)

GC-MS detection has been done on the basis of mass to charge ratio of ionized atoms or molecules to analyze the compounds. It can be identified by the fragmentation pattern and measured by the formation of daughter ions [3]. GC-MS is a standard technique for the detection of VOCs in the expired breath [90]. The characterization of abnormal chemical compounds associated with human diseases has been investigated with the development of gas chromatography (GC) and mass spectroscopy (MS) instruments. Mayakova *et al*. [91] developed a GC-MS method to analyze volatile fatty acids to detect post-operative infections caused by non-clostridial anaerobes in abdominal and gynecological surgery. GC-MS analyses have enabled comprehensive studies and identifications of possible disease markers, these tools are not emerged as regular instruments for clinical diagnosis due to cost effectiveness, laborious and time-consuming sample-preparation methods, and necessities for efficient training and expertise for effective operation and data interpretation [92,93]. Additionally, the high complexity of volatile profiles detected using GC-MS methods prevents the ability to associate these chemical profiles to specific microbial or biotic sources because chemical profiles (unlike aroma profiles) are not considered as a whole in data analyses. These instruments are designed to identify individual chemical compounds, not identify the complex sample mixture as a whole unit. GC-MS instruments have been very useful in facilitating our understanding of the biochemistry of human diseases and disorders, and identifying anomalous VOCs that may serve as diagnostic biomarkers of specific diseases and physiological or genetic disorders, but uses of these instruments should be limited to applications for which they were designed. The limited applicability of GC-MS and similar analytical instruments in clinical diagnoses has prompted the need to develop simpler, cheaper, and more user-friendly diagnostic instruments for routine clinical applications [94].

#### 3.1.2. Gas Chromatography-Flame Ionized Detection (GC-FID)

Sanchez *et al*. [95] developed this GC-FID for exhaled breath analysis. FID is more mass sensitive than concentration sensitive. The FID is a useful general detector for the analysis of organic compounds as it has high sensitivity, large linear response, and low noise. FID is the widely used in GC for the breath test. The changes in mobile phase flow rate do not affect the detector’s response. Phillips and Greenberg [96] examined VOCs in exhaled breath by this technique. The effluent from the column is mixed with hydrogen and air, and ignited. Organic compounds burning in the flame produce ions and electrons that can conduct electricity through the flame. A large electrical potential is applied at the burner tip, and a collector electrode is located above the flame. The current resulting from the pyrolysis of any organic compounds is measured. This method was sensitive, linear, accurate and reproducible. Nevertheless the FID destroys samples through the detection process. They combined a nonpolar dimethyl polysiloxane column and a trifluoropropylmethylpolysiloxane column to achieve adequate selectivity for the VOCs in breath [97,98]. The detection limits of their system are in the low-ppb range [95].

#### 3.1.3. Gas Chromatography-Ion Mobility Spectroscopy (GC-IMS)

**Figure 6:**
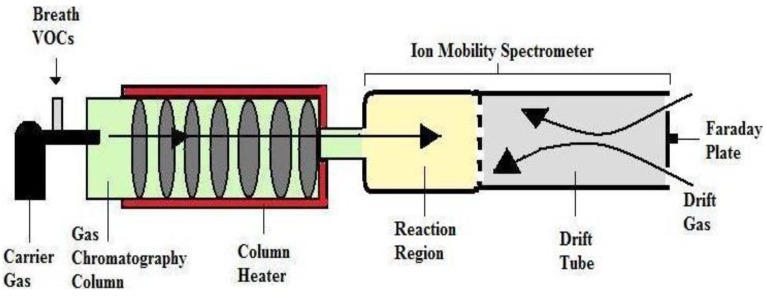
Schematic representation of Gas Chromatography-Ion Mobility Spectroscopy (GC-IMS). Reproduced with permission from [18], copyright 2011, Taylor & Francis.

In 1970s, IMS technique has designed by Cohen and Karase. This technique has developed very quickly [99]. IMS is an effective, simple in practice, and, due to its small size, a very convenient detector. It combines high sensitivity and relatively low cost of a single analysis with a high-speed data acquisition. The time in which is necessary to record a single ion mobility spectrum is in the range of 20–50 ms [100]. The important feature of IMS is that no vacuum is required for its operation. Ambient air can be used as a carrier gas. The IMS detector can be miniaturized and it provides a benefit in commercialization of the system in comparison to other online techniques [101]. IMS has also made great strides towards the analysis of biological materials, such as bacterial spores. The application of a pre-separation technique is helpful for the analysis of complex mixtures. The IMS detector coupled with standard GC columns or multi-capillary columns (MCCs) [102,103]. MCC are characterized by a comparatively high flow rate and high sample capacity in comparison to the single tight columns. The application of MCC enables direct injection of a high gas volume into the column, isothermal separation of volatile organic compounds (VOCs) at the ambient temperature, and multidimensional data analysis of the peaks. The peaks can be identified using chromatographic data (retention times) and specific ion mobility data [104]. The combination of MCC and IMS has been used for breath analysis more and more often. The MCC-IMS technique is supposed to be competitive to GC-MS, which is generally used for breath analysis [105,106,107]. VOCs present in human breath, such as ethanol, acetone, isoprene and other hydrocarbons were measured directly by the MCC-IMS with detection low limits. The application of MCC reduces the negative influence of humidity present in the exhaled breath samples, what improves selectivity of the method [106]. The decrease of the water vapor effect when using MCC improves the sensitivity of determination of the molecules with low proton affinities [107]. The principle method of IMS is to separate ion according to mobility as they travelled through a purified gas in an electric field at atmospheric pressure. This ion travels with varying velocities through the purified gas. The total travel time depends on the drift length, electric field strength; drift gas as air or pure nitrogen, temperature and atmospheric pressure [108]. IMS is a selective detector capable of quantifying substances from mixtures and it is relatively portable and inexpensive. GC-IMS (Figure 6) are two different technologies producing a new system that introduces the advantages of the individual technologies.

Lord and coworkers investigated presence of ethanol and acetone as biological indicator of human health as well as exposure of VOC by GC-IMS [109]. Additionally, the presence of a lot of moisture in the system facilitates cluster formation reactions between the analyzed ions and water molecules. The important advantage of this technique is that the analytes do not need to be pre-concentrated. If the concentration of the analyzed compounds is too high, they are not efficiently ionized since then the amount of reactant ions is insufficient [104]. IMS has shown low sensitivity towards alkanes and benzene-related compounds [110,111].

#### 3.1.4. Gas Chromatography-Electrolyzer Powdered Flame Ionization Detector (GC-EFID)

Common detector is used for detection of many ranges of volatile organic ingredients with a new technique, EFID. The electrolyzer produce oxygen and hydrogen gas mixture for sample separation and is absorbed in the analytical column by this carrier gas [112,113,114].

### 3.2. Proton Transfer Reaction-Mass Spectrometry (PTR-MS)

**Figure 7:**
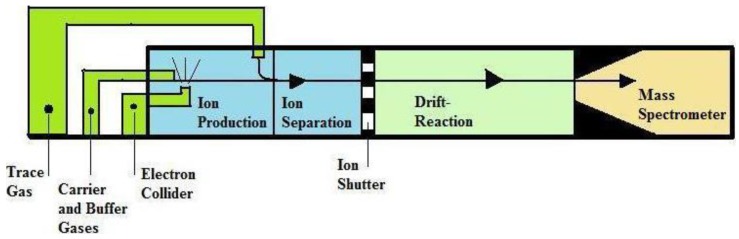
Schematic representation of Proton Transfer Reaction-Mass Spectrometry (PTR-MS), Reproduced with permission from [18], copyright 2011, Taylor & Francis.

PTR-MS (Figure 7), discovered by A. Hansel and coworkers, has been used for the online measurement of complex mixture of trace gas compounds in air with concentration as low as one parts per billion. In PTR-MS, all VOCs have proton affinity higher than H_2_O where each collision proton transfer occurs. PTR-MS has more advantages for breath analysis for complex mixtures of gases because pre-concentration and separation procedure is not required.

If compound gets in higher concentration, like NO_2_, CO_2_, O_2_ and H_2_O, it does not interfere with the measurement; here concentration measurements of very low level up to ppb and frequent/rapid measurements are possible. PTR-MS characterizes the substances individually according to their mass-to-charge ratio and chemical identification provided by other techniques [19,20,22,115,116,117,118,119,120]. Boschetti *et al*. [119] could monitor a large number of VOCs with in limited time period at high sensitivity up to parts per billion. This technique is based on chemical ionization of the target molecules by proton transfer reactions with H_3_O^+^ primary ions. The protonated molecules are accelerated and followed by detection using an inline MS [121]. It includes the fact that samples can be easily analyzed as there are no pre-concentration or separation processes [122]. Moreover, sample analysis with this technique offers very fast response times and even enable real-time measurement of samples in some situations where rapid and sudden changes of VOC concentrations are needed [123]. PTR-MS is a selective technique that dealings only with a limited number of VOCs since it only detects compounds with a proton affinity higher than that of water. PTR-MS cannot differentiate isomeric and isobaric ions as they are all detected at the same nominal mass. It is only used to analyze a relatively small fraction of the total VOC profile present in breath and thus not to define a total breath-print. The chemical identification of the detected ions are required to pin down specific VOCs and/or underlying metabolic processes to exposure or disease status, remains very difficult since no fragmentation is detected. Eventually, this method is not applicable for concentrated samples as the total VOC concentration that can be analyzed should not exceed 10 parts per million per volume (ppmv). This PTR-MS is related only to mass of the product ions but is not a unique marker to identify trace gases; because of the overlap of mass spectra to different isomers it cannot be resolved [120,122].

### 3.3. Selected Ion Flow Tube-Mass Spectrometry (SIFT-MS)

**Figure 8:**
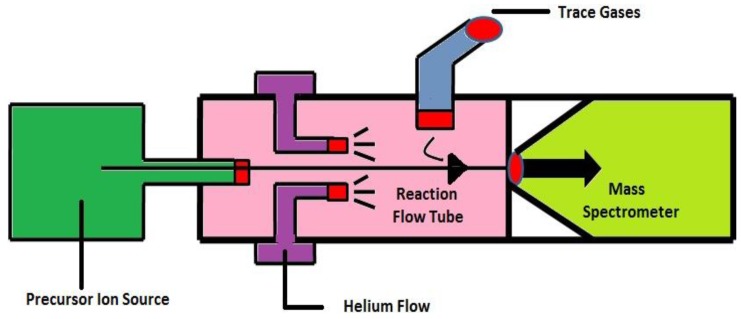
Schematic representation of Selected Ion Flow Tube-Mass Spectrometry (SIFT-MS). Reproduced with permission from [18], copyright 2011, Taylor & Francis.

SIFT-MS (Figure 8) combines fast flow tube technique with quantitative mass spectrometry. It is based on soft chemical ionization of trace gases apart from the major air and breath components in a fast-flowing inert carrier gas by the ion-molecule reactions occur between the trace gases and the preselected precursor ions. These trace gases are detected and quantified with the MS. The SIFT technique has been developed for the detection and quantification of trace gases in breath samples in real time [124]. SIFT-MS is constructed to allow on-line analyses of the exhaled breath, headspace of aqueous liquid and polluted air. The exhaled breath sample is taken into fast flowing inert gas, e.g., helium carrier gas, present as trace gas in the sample and reacts with reagent ions to form specific product ion that identify the compound. SIFT-MS has same feature as other analytical techniques, where the sample collected into bag or onto traps are not required, which can compromise the sample, and time consuming calibration becomes unnecessary. It allows the direct analysis of single exhalations of breath and provides the clinician with immediate results. It is used to analyze the large amount of kinetic data on gas phase ion-neutral reactions [123,124].

Smith *et al*. [123] formulated this SIFT technique as a flow reactor device to perform rapid real-time analysis of the trace gases in the air and in the breath. It can be used to detect many types of trace gases at the sub-ppm level. The major feature of this method is that the time response is very short. The real time fluctuations in a breath sample can also be observed.

### 3.4. Laser Photoacoustic Spectroscopy

**Figure 9:**
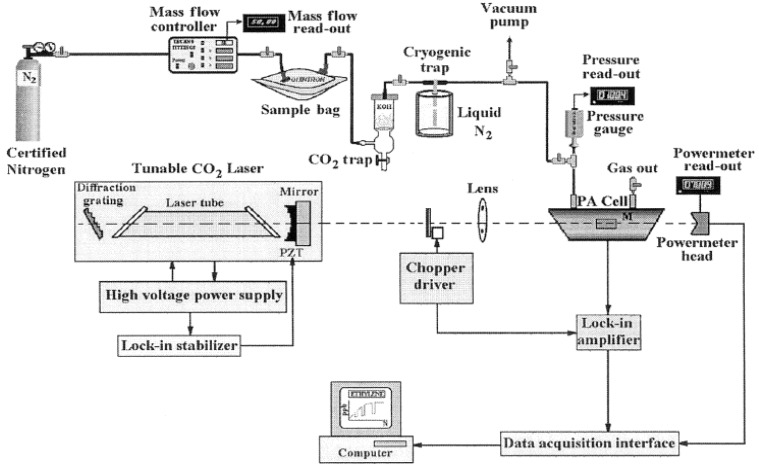
Experimental set up for Laser Photoacoustic Spectroscopy (LPAS), Reproduced with permission from [125]. Copyright 2005, SPIE.

In LPAS technique (Figure 9), home-built, line-tunable and frequency-stabilized CO_2_ laser has been used as a radiation source as its emission spectrum overlaps with the absorption fingerprint of ammonia and ethylene. The laser beam is amplitude modulated by an optical chopper and focused by a ZnSe lens and introduced in the PA cell. The laser power was used to excite the sample gas inside the spectrophone and measured by a two-channel power meter. The laser beam is based on amplitude modulated by using an optical chopper and focused by a ZnSe lens and introduced in the PA cell (spectrophone). The laser power inside the spectrophone is measured by a two-channel power meter. The acoustic waves are formed in the spectrophone and can be detected with four miniature electret microphones which are connected in series. The PA signal is proportional to the trace gas concentration. It is applied to a lock-in amplifier which detects and measures very small single frequency AC signals (usually buried in a larger random noise).

When the PA signal equals the noise voltage given by the background signal the minimum detectable concentration is obtained. Both the mass flow and the total pressure inside the spectrophone are measured with digital instruments. Carbon dioxide makes up approximately five percent of the breath and the CO_2_ laser lines are slightly absorbed by this gas. It is necessary to introduce a trap to remove most of the CO_2_ from the exhaled air. To prevent the undesired supplementary absorption of the interfering gases, especially CO_2_, a KOH based scrubber was introduced before the PA cell. The exhaled samples were collected with a chemically inert aluminized bag. Mouthpiece parts must be replaceable to avoid any risk of transmitting infections from patients to patients. The sample breath has to originate deep in the lungs, since human breath is not a homogeneous gas. To obtain reliable measurements, a gas handling system is introduced. Certified nitrogen is used to transport the sample gas from the sample bag to the PA cell. This LPAS system is able to measure a minimum concentration of 0.2 ppb of ethylene in nitrogen at atmospheric pressure [125].

### 3.5. Chemiluminescence Analyzer

The chemiluminescence analyzer has been adopted from atmospheric NO measurement. It has been used for the detection of NO in human breath. The interest in measuring NO in exhaled breath was not only driven by its potential in disease diagnosis and management, but also by the technology available for NO detection. The chemiluminescence devices are considered as the standard technique and all the later developed detection methods for measuring NO, such as electrochemical and laser based detection. The chemiluminescence analyzers are very sensitive, with detection limits at ppbv-level. Apart from the size and investment cost limitations, the chemiluminescence analyzers suffer from drift. To compensate the drift, frequent calibration is often required for the instrument and it can be used for measurements. Calibration can be done with high NO concentration in the tens of ppmv level or hundreds of ppb [126]. It is an advantageous technique because breath is analyzed directly online to an analyzer or indirectly by sampling of breath in balloon which is analyzed later. Nitric oxide can be measured in breath in part per billion (ppb) [75]. This method is useful for asthmatic patients to detect nitric oxide level. A yearly technical service is recommended by each manufacturer which requires the instrument to be shipped to the dealer or support center. Consequently, the running costs will be increased. These are the limitations for the minimal use of chemiluminescence analyzers in routine clinical applications or home monitoring [51].

### 3.6. Colorimetric Sensor Arrays

Colorimetric sensor arrays can be used in the exhaled breath analysis for detection of compounds. It is especially for the lung cancer patient. Mazzone *et al*. [75] showed that the colorimetric sensor array has 36 spots which contain chemically sensitive compound on disposable cartridges. The changes in color of spots occur due to its contact with volatile active compounds which are present in exhaled breath.

### 3.7. Differential Mobility Spectrometer (DMS)

Differential mobility spectroscopy used in breath analysis for identification of many chemicals on low level concentration as part per billion and diagnosis of many diseases. It is a very sophisticated instrument as compared to traditional GC-MS in all its function [127].

The major expired breath gases along with its concentrations using various analytical techniques are shown in Table 3. Recently, breath analysis methods have been laboratory-based with limited portability for direct use in environmental or occupational conditions [128].

**Table 3:** Techniques and its detection for various gases.

Gases	Techniques	Concentration	References
Ammonia	PTR-MS	90 ppt	[129]
	SIFT-MS	10 ppb	[130]
	GC-IMS	4 ppt	[103]
Acetone	PTR-MS	50 ppb	[131]
		1.33 ± 0.19 ppm	[40]
	GC-MS	0.049 ppb	[40]
Nitrogen Monoxide	Chemiluminescence	200 ppb	[75]
Isoprene	PTR-MS	100 ppb	[40]
	SIFT-MS	0–474 ppb	[132]
Methane	SIFT-MS	0.2 ppm	[133]
Ethanol	PTR-MS	289.00 ± 67.47	[40]
	SIFT-MS	0–1663 ppb	[134]

### 3.8. Nanomaterials for Breath Gas Analysis

**Figure 10:**
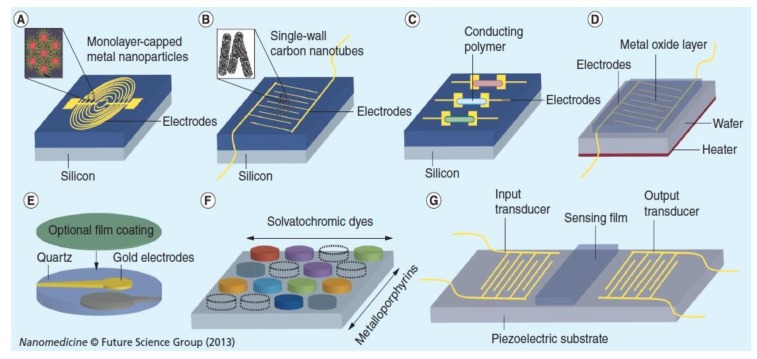
Sensors based on different nanomaterials. (A) Chemiresistors based on monolayer capped metal nanoparticles. (B) Chemiresistors based on single walled carbon nanotubes. (C) Chemiresistors based on conducting polymers. (D) Chemiresistor based on chemicapacitor based on metal oxide film. (E) Quartz microbalance with selective coating. (F) Calorimetric sensor. (G) Surface acoustic wave sensor. Reproduced with permission from [11]. Copyright 2013, Future Medicine.

The implementation of nanotechnology in the field of breath analysis with the chemosensors has been increased in recent years. The nanoscale size makes nanomaterials sensitive to localized entities of similar size, from small molecules to large macromolecules. The nanoscale size of these building blocks provides them with several merits, such as large surface-to-volume ratio and unique chemical, optical, and electrical properties. The increased surface area of the nanomaterials provides highly active interfaces, thus increasing sensitivity and lowering the response and recovery times. Various nanomaterials have been utilized for VOC sensing elements, including nanoparticles and nanowires of different materials and carbon nanotubes. In recent years, special attention has been given to methods incorporating nanomaterial-based gas sensors because they would enable the development of highly sensitive, rapidly responsive, and yet cheap detection systems. For instance, the dynamic range as well as the selectivity of the nanomaterial-based gas sensors can be tailored to accurately detect specific breath VOCs of a given disease [135]. An array of sensors which is made of organically capped gold nanoparticles to detect delicate changes in the exhaled volatile organic compounds associated with acute kidney injury. An array of sensors using gold nanoparticles can be distinguished for the breath of lung cancer patients in an atmosphere of high humidity. Sensors based on the gold nanoparticles forms the origin of an inexpensive and noninvasive diagnostics for the lung cancer patients. It shows great promise for rapid, convenient and cost-effective diagnosis and screening of lung cancer. The developed devices are portable, inexpensive and easier to use in widespread screening and potentially valuable in saving millions of lives every year. This proposed technology will be a considerable saving for both private and public health expenditures. The potential exists for using the proposed technology to diagnose other conditions and diseases, which could mean additional cost reductions and enhanced opportunities to save lives. So far, Chemiresistive gas sensors offer greater usability for portable real time breath sensors due to their miniaturized size, low cost, easy fabrication and simplicity of operation. Recent progress in the synthesis of novel nanostructures has been adopted to develop high sensitivity breathing sensors because it has superior surface area, as well as pore size and distribution. These nanostructures include nanoparticles, hollow spheres, nanostructures such as tubes, wires, and fibers. 1D metal oxide nanostructures have been recognized as one of the most efficient nanoarchitectures for chemiresistive sensors due to their large surface area to volume ratio, high gas accessibility and good thermal stability. Figure 10 shows the various nanomaterials based sensors.

Xu *et al*. [135] prepared the bifunctional magnetic nanoparticles by solid phase extraction method and applied for the analysis of trace amount of aldehydes in human exhaled breath condensate. Six aldehydes were derivatized with 2,4-dinitrophenylhydrazine and formed hydrazones were extracted by the nanoparticles and analyzed by high-performance liquid chromatography-photo diode array detector and provided the low limits of detection (2.9–21.5 nmol·L^−1^), satisfactory reproducibility (2.9%–13.1%) and acceptable recoveries (73.7%–133.1%). Zhen *et al*. [136] demonstrated the nanomaterial based breath test to distinguish the gastric cancer from benign gastric conditions through VOC by GC-MS. They developed the nanomaterial based sensor array using cross reactive and chemically diverse chemiresistors based on two types of nanomaterials namely, organically stabilized spherical gold nanoparticles and single walled carbon nanotubes. The chemical diversity has been achieved through 14 different organic functionalities. Wu *et al*. [137] reported a simple gaseous sensor using ZrO_2_ nanoparticles for the detection of trace-2 propanal using *in situ* enrichment and Cataluminescence (CTL) detection method. The results indicated that the CTL sensor show improved stability and longer term durability with high sensitivity and could potentially be used in trace VOC detection in breath analysis. Marom *et al*. [138] prepared the functionalized gold nanoparticles for the sensor to detect the Chronic Kidney Disease (CKD) and disease progression. It was analyzed by GC-MS. Several substances in the breath were identified which is related with CKD-related biochemical processes or with the accumulation of toxins through kidney function loss. It has been inferred that the breath testing using gold nanoparticle sensors holds future potential as a cost-effective, fast and reliable diagnostic test for early detection of CKD and monitoring of disease progression. Thiol derivatized gold nanoparticles sensors were developed to detect VOCS in the exhaled breath by Jared Stephens. The tested VOCs with this sensor were acetone, ethanol, and the mixture of acetone and ethanol. Sensors were made using several types of thiol derivatized gold nanoparticles by varying two factors such asthiol compound and molar volume loading of the thiol in synthesis. It was reported as several sensors show high selectivity to one or more VOCs [139]. Righettoni *et al*. [140] have demonstrated chemo resistive gas sensor to measure the level of acetone in the exhaled breath and analyzed by PTR-MS. Portable acetone sensors made of 10 mol% Si doped WO_3_ nanoparticles were developed and tested for breath analysis. It has been proven that these sensors were applied to breath acetone monitoring of different test persons and in agreement (>98%) to high-sensitivity. Monolayer Capped Gold Nanoparticles were synthesized by modified two phase method and drop casted onto the inter-digitated gold electrodes. 14 GNP sensors with different organic functionalities were mounted onto a PTFE circuit board to form single array of nanosensors and analyzed by GC-MS. It has been described that the GNP sensor is insensitive to confounding factors and demonstrated for the detection of lung, breast, colorectal, and prostate cancers from exhaled breath by Peng *et al*. [141]. Wang *et al*. [142] were used Cr-WO_3_ nanopowders on a Pt-coated alumina substrate to design the sensor. One or two parallel connected sensors were adhered to a heater. This sensor/heated pair were the key components of portable breath analyzer. Selectivity was done with the various breath gases such as NO, NH_3_, CO and some of the trace gases. Among all the gases acetone shows the better sensitivity. Nanosensor device has been used for breath acetone detection by using the portable breath analyzer. Gouma *et al*. [143] developed a three nanosensor array microsystem for a handheld breath analyzer. Anil Kumar *et al*. [144] developed the array of chemiresistive sensor using gold nanoparticles for the diagnosis of lung cancer and analyzed by GC-MS. Peng *et al*. [145] demonstrated an array of chemiresistors based on functionalized gold nanoparticles to distinguish between the breath of lung cancer patients and healthy controls without the need for dehumidification or pre-concentration of the lung cancer biomarkers. Gouma and Stanacevic reported a novel concept for a three nanosensor array microsystem for the handheld breath analyzer. Their work was based on a metal oxide nanosensor with three distinct temperatures and interfaced the sensor array into an integrated circuit with temperature controls. It was tested with a single exhaled breath and analyzing was done with pertain to ammonia, isoprene and carbon dioxide gases [146]. Broza *et al*. [147] demonstrated nanomaterial based breath test for the short-term follow-up after lung tumor resection. Nanomaterial-based (Au NP and Pt NP) sensor were fabricated to form the sensor-reservoir and GC-MS. Righettoni *et al*. developed the chemo-resistive detector using pure and Si-doped WO_3_ nanoparticles for quantitative analysis of acetone concentration in the breath. It has been investigated as a function of temperature (325–500 °C), 90% Relative Humidity (RH) and interfering analyte concentration. These solid state detectors offer a portable and cost effective alternative to more bulky systems for noninvasive diabetes detection by human breath analysis [148]. Lonescu *et al*. [149] developed a cross-reactive array based on bilayers of single-wall carbon nanotubes and designed polycyclic aromatic hydrocarbon derivatives with various aromatic coronae and side groups. They demonstrated the sensor array to discriminate between VOCs which is associated with multiple sclerosis (tentatively, hexanal and 5-methyl-undecane). It was shown that multiple sclerosis can be identified by breath analysis through the selective and more suitable polycyclic aromatic hydrocarbons derivatives. Gruber *et al*. [150] presented a pilot study that demonstrates the feasibility of VOC profiling for identifying head and neck Squamous cell carcinoma (HNSCC). Three compounds were identified as possible markers for HNSCC and/or benign lesions of the head and neck: ethanol, 2-propenenitrile and undecane. They demonstrated the discriminative power of a nanomaterial-based sensor array. Breath prints derived from the sensory output provided allowed distinguishing HNSCC from benign tumors and from healthy states. The results shown could eventually lead to the development of a simple breath test that may be used to aid and complement conventional HNSCC diagnosis. Bachar *et al*. [151] presented a comparative study of the VOCs sensing performance of Polycyclic aromatic hydrocarbon (PAH) derivatives with different types of side chains as sensing elements in arrays of chemiresistors and Quartz crystal Microbalance (QCM) sensors. The PAH derivatives provided good sensitivity and selectivity towards different polar and nonpolar VOCs from the families of alcohols, alkanes, ethers and aromatics under extremely varying humidity conditions (5%–80% RH).They demonstrated that the chemiresistor arrays performed superior at controlled RH levels, especially in dry atmospheres, whereas the QCM arrays were ideally suited for applications under extremely variable background humidity. Fu *et al*. [152] developed a silicon microreactor coated with 2-(aminooxy)-*N*,*N*,*N*-trimethylethanammonium (ATM) which have been identified four carbonyl VOCs in exhaled breath. In elevated concentrations, it could diagnose lung cancer. The concentrations of 2-butanone, 3-hydroxy-2-butanone, 2-hydroxyacetaldehye, and (4-hydroxyhexenal) 4-HHE in breath were determined by FT-ICRMS analysis of the respective ATM-VOC adducts, and elevated concentrations of these adducts relative to concentrations in healthy patients, or even patients with benign pulmonary nodules which indicates the presence of lung cancer. The concentration of 2-butanone can be used to distinguish stage I lung cancer from stages II through IV. Furthermore, the concentration of 4-HHE may be used to distinguish squamous cell carcinoma from adenocarcinoma and other one—small cell lung cancer (NSCLC), and the concentrations of (4-hydroxynonenal) 4-HNE and C_5_H_10_O can be used to distinguish small cell lung cancer (SCLC) patients from NSCLC patients. These have immediate application as an accurate, noninvasive means for the diagnosis of lung cancer. Gouma and Kalyanasundaram reported on a monoclinic tungsten trioxide (WO3) nanostructured probe for selective detection of minute NO concentrations in the presence of interfering VOCs, such as ethanol, methanol, isoprene, acetone and CO [153].

Table 4 shows the summary of different materials based sensors and their applications in the field of breath analysis.

**Table 4:** Materials and its monitoring techniques in the field of breath analysis.

Materials	Analytical Technology/Device	Gases	Reference
Gold nanoparticles	GC-MS	VOCs	[144]
MoO_3_ Nanosensor	Nanosensor device	Isoprene, CO_2_, NH_3_	[143]
Gold Nanoparticles	GC-MS	VOCs	[141]
Si-doped WO_3_ Nanoparticle	Chemiresistor/PTR-MS	Acetone	[140]
Tungsten trioxide (WO3) nanostructured probe	-	ethanol, methanol, isoprene, acetone	[153]
Gold Nanoparticles and Single Walled Carbon Nanotubes	Chemiresistor	VOCs	[136]
Functionalized gold nanoparticles	GC-MS	VOCs	[138]
Thiol derivatized gold nanoparticles sensors	GC-MS	Acetone and Ethanol	[139]
Nanomaterial-based (Au NP and Pt NP) sensor	GC-MS	VOCs	[148]
Chitosan	Chemiresistor	Acetone	[154]
Gold nanoparticles decorated polyaniline	Chemiresistor	VSCs	[155]
In_2_O_3_ and Pt-In_2_O_3_	Chemiresistor	Acetone	[156]
PEDOT:PSS coated nanofibrous TiO_2_	Chemiresistor	Nitric Oxide	[157]
MoO_3_	Chemiresistor	Ammonia	[147]
H_2_SO_4_ solution	Chemiresistor	Ammonia	[158]
MWCNTs	Chemiresistor	Sleep apnea	[159]
Chemically functionalized carbon nanotubes	Chemiresistor	Nitric Oxide	[160]
Hemitubes of Pt-WO_3_	Chemiresistor	Acetone	[161]

## 4. Conclusions

The discovery of novel technologies and biomarkers to detect the diseases has been increasing persistently. There are sophisticated analytical methods for disease detection and they are currently used in well-trained clinical and professional laboratories. The aim is to achieve fast and inexpensive personalized medicine that could be implemented globally, even in developing countries. The integration of nanoscale medical technologies into this scaffold will be highly desirable and allow high-speed global diagnostics. This review reports on technologies for expired breath gas analysis. Exhaled breath monitoring techniques have been discussed along with the applications of nanotechnology for clinical diagnostics.

## References

[1] Bhairavi P. Exhaled breath analysis a review of clinical applications to determine oxidative stress. http://www.researchgate.net/publication/265149864_Exhaled_Breath_Analysis_A_Review_of_Clinical_Applications_to_Determine_Oxidative_Stress.

[2] Popov T.A. (2011). Human exhaled breath analysis. Ann. Allergy Asthma Immunol..

[3] Cheng W.-H., Le W.-J. (1999). Technology development in breath microanalysis for clinical diagnosis. J. Lab. Clin. Med..

[4] Buszewski B., Kesy M., Ligor T., Amann A. (2007). Human exhaled air analytics: Biomarkers of diseases. Biomed. Chromatogr..

[5] Braun P.X., Gmachl C.F., Dweik R.A. (2012). Bridging the collaborative gap: Realizing the clinical potential of breath analysis for disease diagnosis and monitoring-tutorial. IEEE Sens..

[6] Murtz M. (2005). Breath diagnostics using laser spectroscopy. Opt. Photonics News.

[7] Monk P.S., Willis K.A. (2010). Breath analysis. Educ. Chem..

[8] Gouma P. (2011). Selective oxide sensors as non-invasive disease monitors. SPIE Newsroom.

[9] Haick H., Broza Y.Y., Mochalski P., Ruzsanyi V., Amann A. (2014). Assessment, origin, and implementation of breath volatile cancer markers. Chem. Soc. Rev..

[10] Anton A., Pawel M., Vera R., Yoav Y.B., Hossam H. (2014). Assessment of the exhalation kinetics of volatile cancer biomarkers based on their physicochemical properties. J. Breath Res..

[11] Broza Y.Y., Haick H. (2013). Nanomaterial-based sensors for detection of disease by volatile organic compounds. Nanomedicine.

[12] Hakim M., Broza Y.Y., Barash O., Peled N., Phillips M., Amann A., Haick H. (2012). Volatile Organic Compounds of Lung Cancer and Possible Biochemical Pathways. Chem. Rev..

[13] Konvalina G., Haick H. (2013). Sensors for breath testing: From nanomaterials to comprehensive disease detection. Acc. Chem. Res..

[14] Shaji J., Jadhav D. (2010). Breath biomarker for clinical diagnosis and different analysis technique. Res. J. Pharm. Biol. Chem. Sci..

[15] Vereb H., Dietrich A.M., Alfeeli B., Agah M. (2011). The possibilities will take your breath away: Breath analysis for assessing environmental exposure. Environ. Sci. Technol..

[16] Gouma P., Stanacevic M. (2011). Selective nanosensor array microsystem for exhaled breath analysis. Procedia Eng..

[17] Cao W., Duan Y. (2006). Breath analysis: Potential for clinical diagnosis and exposure assessment. Clin. Chem..

[18] Hibbard T., Killard A.J. (2011). Breath ammonia analysis: Clinical application and measurement. Breath Ammon. Clin. App. Meas..

[19] Lindinger W., Hansel A. (1997). Analysis of trace gases at ppb levels by proton transfer reaction mass spectrometry (PTR-MS). Plasma Sources Sci. Technol..

[20] Lindinger W., Hansel A., Jordan A. (1998). On-line monitoring of volatile organic compounds at pptv levels by means of proton-transfer-reaction mass spectrometry (PTR-MS) medical applications, food control and environmental research. J. Mass Spect. Ion Proc..

[21] Schmidt F.M., Vaittinen O., Metsälä M., Lehto M., Forsblom C., Groop P.H., Halonen L. (2013). Ammonia in breath and emitted from skin. J. Breath Res..

[22] Warneke C., Kuczynski J., Hansel A., Jordan A., Vogel W., Lindinger W. (1996). Proton transfer reaction mass spectrometry (PTR-MS): Propanol in human breath. J. Mass Spect. Ion Proc..

[23] Galassetti P.R., Novak B., Nemet D., Rose-Gottron C., Cooper D.M., Meinardi S., Newcomb R., Zaldivar F., Blake D.R. (2005). Breath ethanol and acetone as indicators of serum glucose levels: An initial report. Diabetes Technol. Ther..

[24] Perez-Guaita D., Kokoric V., Wilk A., Garrigues S., Mizaikoff B. (2014). Towards the determination of isoprene in human breath using substrate-integrated hollow waveguide mid-infrared sensors. J. Breath Res..

[25] Berg J.M., Tymoczko J.L., Stryer L. (2002). Protein turnover and amino acid catabolism. Biochemistry.

[26] Voet D., Voet J.G., Pratt C.A. (1999). The urea cycle. Fundamentals of Biochemistry.

[27] Tate P. (2009). Protein Metabolism. Seeley’s Principles of Anatomy and Physiology.

[28] Essiet I.O. (2013). Diagnosis of kidney failure by analysis of the concentration of ammonia in exhaled human breath. J. Emerg. Trends Eng. Appl. Sci..

[29] Butterworth R.F. (2003). Hepatic encephalopathy. Alcohol Res. Health.

[30] National Digestive Diseases Clearing House. http://digestive.niddk.nih.gov/diseases/pubs/hpylori/index.htm.

[31] Society of Nuclear Medicine Procedure Guideline for C-14 Urea Breath Test. http://interactive.snm.org/docs/pg_ch07_0403.pdf.

[32] Kearney D.J., Hubbard T., Putnam D. (2002). Breath ammonia measurement in *Helicobacter pylori* infection. Dig. Dis. Sci..

[33] Amano A. (2002). Monitoring ammonia to assess halitosis. Oral Surg. Oral Med. Oral Pathol..

[34] Van den Broek A.M., Feenstra L., de Baat C. (2007). A review of the current literature on aetiology and measurement methods of halitosis. J. Dent..

[35] MacGregor G. (2005). Breath condensate ammonium is lower in children with chronic asthma. Eur. Respir. J..

[36] Nilsson A. Development of a handheld meter for monitoring of diabetes using exhaled air. http://www.tekniskdesign.se/download/Rapport_Exjobb_-_AndersNilsson_-_INLAGA.pdf.

[37] Crofford O.B., Mallard R.E., Winton R.E., Rogers N.L., Jackson C., Keller U. (1977). Acetone in Breath and blood. Trans. Am. Clin. Climatol. Assoc..

[38] Reichard G.A., Skutches C.L., Hoeldtke R.D., Owen E.O. (1986). Acetone metabolism in humans during diabetic ketoacidosis. Diabetes.

[39] Deng C., Zhang J., Yu X., Zhang W., Zhang X. (2004). Determination of acetone in human breath by gas chromatography-mass spectrometry and solid-phase microextraction with on-fiber derivatization. J. Chromatogr..

[40] Massick S. Portable Breath Acetone Measurements Combine Chemistry and Spectroscopy.

[41] Toyooka T., Hiyama S., Yamada Y. (2013). A prototype portable breath acetone analyzer for monitoring fat loss. J. Breath Res..

[42] King J., Koc H., Unterkoflera K., Mochalski P., Kupferthalera A., Teschl G., Teschl S., Hinterhuber H., Amanna A. (2010). Physiological modeling of isoprene dynamics in exhaled breath. J. Theoret. Biol..

[43] Mendis S., Sobotka P.A., Euler D.E. (1995). Expired hydrocarbons in patients with acute myocardinal infarction. Free Rad. Res..

[44] McGrath L.T., Patrick R., Silke B. (2001). Breath isoprene in patients with heart failure. Eur. J. Heart Fail..

[45] Bajtarevic A., Ager C., Pienz M., Klieber M., Schwarz K., Ligor M., Ligor T., Filipiak W., Denz H., Fiegl M. (2009). Noninvasive detection of lung cancer by analysis of exhaled breath. BMC Cancer.

[46] Kinoyama M., Nitta H., Ohta T., Hatase Y., Hara S., Hirakawa K., Watanabe K., Watanabe A., Ueda H. (2006). Diurnal variation in the concentration of methane in the breath of methane producers. Microb. Ecol. Health Dis..

[47] De Lacy Costelo J.P.B., Ledochowski M., Ratcliffe M.N. (2013). The importance of methane breath. testing: A review. J. Breath Res..

[48] Tuboly E., Szabo A., Eros G., Mohacsi A., Szabo G., Tengölics R., Rakhely G., Boros M. (2013). Determination of endogenous methane formation by photoacoustic spectroscopy. J. Breath Res..

[49] Toombs C.F., Insko M.A., Wintner E.A., Deckwerth T.L., Usansky H., Jamil K., Goldstein B., Cooreman M., Szabo C. (2010). Detection of exhaled hydrogen sulphide gas in healthy human volunteers during intravenous administration of sodium sulphide. Br. J. Clin. Pharmacol..

[50] Wu R., Yao W.Z., Chen Y.H., Geng B., Tang C.S. (2008). Plasma level of endogenous hydrogen sulfide in patients with acute asthma. Beijing Da Xue Xue Bao.

[51] Chen Y.H., Yao W.Z., Gao J.Z., Geng B., Wang P.P., Tang C.S. (2009). Serum hydrogen sulfide as a novel marker predicting bacterial involvement in patients with community-acquired lower respiratory tract infections. Respirology.

[52] Wang P., Zhang G., Wondimu T., Ross B., Wang R. (2014). Hydrogen sulfide and asthma. Exp. Physiol..

[53] Bernard C., Tripier A. Leconssur les effets des substances toxiques et medicamenteuses. http://gallica.bnf.fr/ark:/12148/bpt6k773289.

[54] Furne J., Saeed A., Levitt M.D. (2008). Levitt Whole tissue hydrogen sulfide concentrations are orders of magnitude lower than presently accepted values. Am. J. Physiol. Regul. Integr. Comp. Physiol..

[55] Rosenberg M., McCulloch C.A. (1992). Measurement of oral malodor: Current methods and future prospects. J. Periodontol..

[56] Suarez F.L., Furne K.J., Springfield J., Levitt D.M. (2000). Morning breath odor: Influence of treatments on sulfur gases. J. Dent. Res..

[57] Miller D.L., Roth M.B. (2007). Hydrogen sulfide increases thermotolerance and lifespan in *Caenorhabditis elegans*. Proc. Natl. Acad. Sci. USA.

[58] Blackstone E., Roth M.B. (2007). Suspended animation-like state protects mice from lethal hypoxia. Shock.

[59] Rosenberg M. (1996). Clinical assessment of bad breath: Current concepts. J. Am. Dent. Assoc..

[60] Lundberg J.O., Weitzberg E., Lundberg J.M., Alving K. (1996). Nitric oxide in exhaled air. Eur. Respir. J..

[61] Gustafsson L.E., Leone A.M., Persson M.G., Wiklund N.P., Moncada S. (1991). Endogenous nitric oxide is present in the exhaled air of rabbits, guinea pigs and humans. Biochem. Biophys. Res. Commun..

[62] Barnes P.J. (1995). Nitric oxide and airway disease. Ann. Med..

[63] Archer S. (1993). Measurement of nitric oxide in biological models. FASEB J..

[64] Saleh D., Ernst P., Lim S., Barnes P.J., Giaid A. (1998). Increased formation of the potent oxidant peroxynitrite in the airways of asthmatic patients is associated with induction of nitric oxide synthase: Effect of inhaled glucocorticoid. FASEB J..

[65] Exhaled nitric oxide in patients with asthma: association with NOS1 genotype. http://www.aai.mf.vu.lt/alerimun/bibliografija/arch_2001/aai2.pdf.

[66] Pavord I.D., Shaw D.E., Gibson P.G., Taylor D.R. (2008). Inflammometry to assess airway diseases. Lancet.

[67] Kharitonov S.A., Yates D., Barnes P.J. (1995). Increased nitric oxide in exhaled air of normal human subjects with upper respiratory tract infections. Eur. Respir. J..

[68] Barnes P.J., Kharitonov S.A. (1996). Exhaled nitric oxide: A new lung function test. Thorax.

[69] Maziak W., Loukides S., Culpitt S., Sullivan P., Kharitonov S.A., Barnes P.J. (1998). Exhaled nitric oxide in chronic obstructive pulmonary disease. Am. J. Respir. Crit. Care Med..

[70] Dotsch J., Demirakca S., Terbrack H.G., Huls G., Rascher W., Kuhl P.G. (1996). Airway nitric oxide in asthmatic children and patients with cystic fibrosis. Eur. Respir. J..

[71] Riely C.A., Cohen G., Lieberman M. (1974). Ethane evolution: A new index of lipid peroxidation. Science.

[72] Handelman G.J., Rosales L.M., Barbato D., Luscher J., Adhikarla R., Nicolosi R.J., Finkelstein F.O., Ronco C., Kaysen G.A., Hoenich N.A. (2003). Breath ethane in dialysis patients and control subjects. Free Radic. Biol. Med..

[73] Breiman L. (2001). Random forests. Mach. Learn..

[74] Efron B. (1979). Bootstrap methods: Another look at the jackknife. Ann. Stat..

[75] Mazzone P.J., Hammel J., Dweik R., Na J., Czich C., Laskowski D., Mekhail T. (2007). Diagnosis of lung cancer by the analysis of exhaled breath with a colorimetric sensor array. Thorax.

[76] Shawe-Taylor J., Cristianini N. (2004). Kernel Methods for Pattern Analysis.

[77] Guyon I., Weston J., Barnhill S. (2002). Gene selection for cancer classification using support vector machine. Mach. Learn..

[78] Paredi P., Kharitonov S.A., Leak D., Ward S., Cramer D., Barnes P.J. (2000). Exhaled ethane, a marker of lipid peroxidation, is elevated in chronic obstructive pulmonary disease. Am. J. Respir. Crit. Care Med..

[79] Dillard C.J., Tappel A.L. (1989). Lipid peroxidation products in biological tissues. Free Radic. Biol. Med..

[80] Knutson M.D., Handelman G.J., Viteri F.E. (2000). Methods for measuring ethane and pentane in expired air from rats and humans. Free Radic. Biol. Med..

[81] Scotter J.M., Allardyce R.A., Langford V.S., Hill A., Murdoch D.R. (2006). The rapid evaluation of bacterial growth in blood cultures by selected ion flow tube-mass spectrometry (SIFT-MS) and comparison with the BacT/ALERT automated blood culture system. J. Microbiol. Methods.

[82] Thorn R.M., Reynolds D.M., Greenman J. (2011). Multivariate analysis of bacterial volatile compound profiles for discrimination between selected species and strains *in vitro*. J. Microbiol. Methods.

[83] Allardyce R.A., Hill A.L., Murdoch D.R. (2006). The rapid evaluation of bacterial growth and antibiotic susceptibility in blood cultures by selected ion flow tube mass spectrometry. Diagn. Microbiol. Infect. Dis..

[84] Wendland B.E., Aghdassi E., Tam C., Carrrier J., Steinhart A.H., Wolman S.L., Baron D., Allard J.P. (2001). Lipid peroxidation and plasma antioxidant micronutrients in Crohn’s disease. Am. J. Clin. Nutr..

[85] Olopade C.O., Christon J.A., Zakkar M., Hua C., Swedler W.I., Scheff P.A., Rubinstein I. (1997). Exhaled pentane and nitric oxide levels in patients with obstructive sleep apnea. Chest.

[86] Hietanen E., Bartsch H., Bereziat J.C., Camus A.M., McClinton S., Eremin O., Davidson L., Boyle P. (1994). Diet and oxidative stress in breast, colon and prostate cancer patients: A case-control study. Eur. J. Clin. Nutr..

[87] Mendis S., Sobotka P.A., Leja F.L., Euler D.E. (1995). Breath pentane and plasma lipid peroxides in ischemic heart disease. Free Radic. Biol. Med..

[88] Dweik R.A. (2008). Exhaled breath analysis: The new frontier in medical testing. J. Breath Res..

[89] Ghoos Y., Hiele M., Rutgeerts P., Vantrappen G. (1989). Porous-layer open-tubular gas chromatography in combination with an ion trap detector to assess volatile metabolites in human breath. Biomed. Environ. Mass Spectrom..

[90] Giardina M., Olesik S.V. (2003). Application of low-temperature glassy carbon-coated macrofibers for solid-phase microextraction analysis of simulated breath volatiles. Anal. Chem..

[91] Mayakova T.I., Kuznetsova E.E., Lazareva M.V., Dolgushina G.S. (1989). Quantitative estimation of volatile fatty acids by means of gas chromatography in rapid diagnosis of non-clostridial anaerobic infection. Voprosy Meditsinskoi Khimii.

[92] Manolis A. (1983). The diagnostic potential of breath analysis. Clin. Chem..

[93] Phillips M. (1997). Method for the collection and assay of volatile organic compounds in breath. Anal. Biochem..

[94] Wilson A.D., Baietto M. (2011). Advances in electronic-nose technologies developed for biomedical applications. Sensors.

[95] Sanchez J.M., Sacks R.D. (2003). GC analysis of human breath with a series-coupled column ensemble and a multibed sorption trap. Anal. Chem..

[96] Phillips M., Greenberg J. (1991). Method for the collection and analysis of volatile compounds in the breath. J. Chromatogr. Biomed. Appl..

[97] Mendis S., Sobotka P.A., Euler D.E. (1994). Pentane and isoprene in expired air from humans: Gas-chromatographic analysis of single breath. Clin. Chem..

[98] Kneepkens C.M.E., Lepage G., Roy C.C. (1994). The potential of the hydrocarbon breath test as a measure of lipid peroxidation. Free Radic. Biol. Med..

[99] Cohen M.J., Karasek F.W. (1970). Plasma Chromatography—A new dimension for gas chromatography and mass spectrometry. J. Chromatogr. Sci..

[100] Baumbach J.I. (2006). Process analysis using ion mobility spectrometry. Anal. Bioanal. Chem..

[101] Pfeifera K.B., Sanchez R.C. (2002). Miniaturized ion mobility spectrometer system for explosives and contraband detection. Int. J. Ion Mobil. Spectrom..

[102] Snyder A.P., Maswadeh W.M., Eiceman G.A., Wang Y.F., Bell S.E. (1995). Multivariate statistical analysis characterization of application-based ion mobility spectra. Anal. Chim. Acta.

[103] Ruzsanyi V., Baumbach J.I., Sielemann S., Litterst P., Westhoff M., Freitag L. (2005). Detection of human metabolites using multi-capillary columns coupled to ion mobility spectrometers. J. Chromatogr. A.

[104] Baumbach J.I., Westhoff M. (2006). Ion mobility spectrometry to detect lung cancer and airway infections. Spectrosc. Eur..

[105] Ruzsanyi V., Sielemann S., Baumbach J.I. (2002). Determination of VOCs in human breath using IMS. Int. J. Ion Mobil. Spectrom..

[106] Ligor T., Szeliga J., Jackowski M., Buszewski B. (2007). Preliminary study of volatile organic compounds from breath and stomach tissue by means of solid phase microextraction and gas chromatography-mass spectrometry. J. Breath Res..

[107] Vautz W., Ruszany V., Sielemann S., Baumbach J.I. (2004). Sensitive ion mobility spectrometry of humid ambient air using 10.6 eV UV-IMS. Int. J. Ion Mobil. Spectrom..

[108] Haley L.V., Romeskie J.M. Development of an explosives detection system using fast GC-IMS technology. Proceedings of the IEEE 32nd Annual 1998 International Carnahan Conference on Security Technology.

[109] Lord H., Yu Y.F., Segal A., Pawliszyn J. (2002). Breath analysis and monitoring by membrane extraction with sorbent interface. Anal. Chem..

[110] Borsdorf H., Schelhorn H., Flachowsky J., Doring H.-R., Stach J. (2000). Corona discharge ion mobility spectrometry of aliphatic and aromatic hydrocarbons. Int. J. Ion Mobil. Spectrom..

[111] Eiceman G.A., Nazarov E.G., Rodriguez J.E., Bergloff J.F. (1998). Positive reactant ion chemistry for analytical, high temperature ion mobility spectrometry (IMS): Effects of electric field of the drift tube and moisture, temperature, and flow of the drift gas. Int. J. Ion Mobil. Spectrom..

[112] Amirav A. (1997). Flame-based Method and Apparatus for Analyzing a Sample.

[113] Amirav A., Tzanani N. (2000). Electrolyzer Device and Method for the Operation of Flame Ionization Detectors. U.S. Patent.

[114] Tzanani N., Amirav A. (1997). Electrolyzer-powered flame ionization detector. Anal. Chem.

[115] Hansel A., Jordan A., Holzinger R., Prazeller P., Vogel W., Lindinger W. (1995). Proton transfer reaction mass spectrometry: On-line trace analysis at the ppb level. J. Mass Spect. Ion Proc..

[116] Jordan A., Hansel A., Holzinger R., Lindinger W. (1995). Acetonitrile and benzene in the breath of smokers and non-smokers investigated by proton transfer reaction mass spectrometry. J. Mass Spect. Ion Proc..

[117] Taucher J., Hansel A., Jordan A., Lindinger W. (1996). Analysis of compounds in human breath after ingestion of garlic using proton-transfer-reaction mass spectrometry. J. Agric. Food Chem..

[118] Amann A., Poupart G., Telser S., Ledochowski M., Schmid A., Mechtcheriakov S. (2004). Applications of breath gas analysis in medicine. Int. J. Mass Spect..

[119] Boschetti A., Biasioli F., Opbergen M., Warneke C., Jordan A., Holzinger R., Prazeller P., Karl T., Hansel A., Lindinger W. (1999). PTR-MS real time monitoring of the volatile organic compounds during postharvest aging of berryfruit postharvest biology and technology. Postharvest Biol. Technol..

[120] Blake R.S., Whyte C., Hughes C.O., Ellis A.M., Monks P.S. (2004). Demonstration of proton-transfer reaction time-of-flight mass spectrometry for real-time analysis of trace volatile organic compounds. Anal. Chem..

[121] Hewitt C.N., Hayward S., Tani A. (2003). The application of proton transfer reaction-mass spectrometry (PTR-MS) to the monitoring and analysis of volatile organic compounds in the atmosphere. J. Environ. Monit..

[122] Smith D., Spanel P. (1996). The novel selected ion flow tube approach to trace gas analysis of air and breath. Rapid Commun. Mass Spectrum..

[123] Smith D., Spaněl P. (2005). Selected ion flow tube mass spectrometry (SIFT flow tube approach to trace gas analysis. Mass Spectrom. Rev..

[124] Dumitrasa C.D., Giubileob G., Puiu A. (2004). Investigation of human biomarkers in exhaled breath by laser photoacoustic spectroscopy. Proc. SPIE.

[125] Cristescu M.S., Mandon J., Harren M.J.F., Merilainen P., Ogman M.H. (2013). Methods of NO detection in exhaled breath. J. Breath Res..

[126] Lundberg J., Rinder J., Weitzberg E., Lundberg J.M., Alving K. (1994). Nasally exhaled nitric oxide in humans originates mainly in the paranasal sinuses. Acta Physiol. Scand..

[127] Sankaran S., Zhao W., Loyola B., Morgan J., Molina M., Shivo M., Rana R., Kenyon N., Davis C. Microfabricated differential mobility spectrometers for breath analysis. Proceedings of the 2007 IEEE Sensors.

[128] Tisch U., Haick H. (2010). Nanomaterials for cross-reactive sensor arrays. MRS Bull..

[129] Norman M., Spirig C., Wolff V., Trebs I., Flechard C., Wisthaler A., Schnitzhofer R., Hansel A., Neftel A. (2009). Intercomparison of ammonia measurement techniques at an intensively managed grassland site (Oensingen, Switzerland). Atmos. Chem. Phys..

[130] Davies S., Spanel P., Smith D. (1997). Quantitative analysis of ammonia on the breath of patients in end-stage renal failure. Kidney Int..

[131] King J., Kupferthaler A., Unterkofler K., Koc H., Teschl S., Teschl G., Miekisch W., Schubert A., Hinterhuber H., Amann A. (2009). Isoprene and acetone concentration profiles during exercise on an ergometer. J. Breath Res..

[132] Dummer J.F. (2010). Analysis of Volatile Biomarkers of Airway Inflammation in Breath. Ph.D. Thesis.

[133] Madasamy T., Pandiaraj M., Balamurugan M., Karnewar S., Benjamin A.R., Venkatesh K.A., Vairamani K., Kotamraju S., Karunakaran C. (2012). Virtual electrochemical nitric oxide analyzer using copper, zinc superoxide dismutase immobilized on carbon nanotubes in polypyrrole matrix. Talanta.

[134] Dryahina K., Smith D., Spanel P. (2010). Quantification of methane in humid air and exhaled breath using selected ion flow tube mass spectrometry. Rapid Commun. Mass Spectrom..

[135] Xu H., Wei Y., Zhu L., Huang J., Li Y., Liu F., Wang S., Liu S. (2014). Bifunctional magnetic nanoparticles for analysis of aldehyde metabolites in exhaled breath of lung cancer patients. J. Chrmotogr..

[136] Zhen-qin X., Broza Y.Y., Ionsecu R., Tisch U., Ding L., Liu H., Song Q., Pan Y.Y., Xiong F.X., Gu K.S. (2013). A nanomaterial-based breath test for distinguishing gastric cancer from benign gastric conditions. Br. J. Cancer.

[137] Wu Y., Wen F., Liu D., Kong H., Zhanga C., Zhang S. (2011). Analysis of 2-propanol in exhaled breath using *in situ* enrichment and cataluminescence detection. J. Biol. Chem. Lumin..

[138] Marom O., Nakhoul F., Tisch U., Shiban A., Abassi Z., Haick H. (2012). Gold nanoparticle sensors for detecting chronic kidney disease and disease progression. Nanomedicine.

[139] Stephens J.S. Investigation of Thiol Derivatized Gold Nanoparticle Sensors for Gas Analysis. http://digital.library.louisville.edu/cdm/singleitem/collection/etd/id/2729/rec/2.

[140] Righettoni M., Tricoli A., Gass S., Schmid A., Anton A., Pratsinis S.E. (2012). Breath acetone monitoring by portable Si:WO_3_ gas sensors. Anal. Chim. Acta.

[141] Peng G., Hakim M., Broza Y.Y., Billan S., Abdah-Bortnyak R., Kuten A., Tisch U., Haick H (2010). Detection of lung, breast, colorectal, and prostate cancers from exhaled breath using a single array of nanosensors. Br. J. Cancer.

[142] Wang L., Kalyanasundaram K., Stanacevic M., Gouma P. (2010). Nanosensor device for breath acetone detection. Sens. Lett..

[143] Gouma P., Prasad A., Stanacevic S. (2011). A selective nanosensor device for exhaled breath analysis. J. Breath Res..

[144] Kumar A., Boruah B.M., Liang X.-J. (2011). Gold Nanoparticles: Promising nanomaterials for the diagnosis of cancer and HIV/AIDS. J. Nanomater..

[145] Peng G., Tisch U., Adams O., Hakim M., Shehada N., Broza Y., Billan S., Abdah-Bortnyak R., Kuten A., Haick H. (2009). Diagnosing lung cancer in exhaled breath using gold nanoparticles. Nat. Nanotechnol..

[146] Gouma P., Kalyanasundaram K., Wang L., Yun X., Stanacevic M. (2010). Nanosensor and breath analyzer for ammonia detection in exhaled human breath. IEEE Sens..

[147] Broza Y.Y., Kremer R., Tisch U., Gevorkyan A., Shiban A., Best L.A., Haick H. (2013). A nanomaterial-based breath test for short-term follow-up after lung tumor resection. Nanomed. Nanotechnol. Biol. Med..

[148] Righettoni M., Tricoli A., Pratsinis S.E. (2010). Si:WO_3_ sensors for highly selective detection of acetone for easy diagnosis of diabetes by breath analysis. Anal. Chem..

[149] Ionescu R., Broza Y., Shaltieli H., Sadeh D., Zilberman Y., Feng X., Glass-Marmor L., Lejbkowicz I., Müllen K., Miller A. (2011). Detection of multiple sclerosis from exhaled breath using bilayers of polycyclic aromatic hydrocarbons and single-wall carbon nanotubes. ACS Chem. Neurosci..

[150] Gruber M., Tisch U., Jeries R., Amal H., Hakim M., Ronen O., Marshak T., Zimmerman D., Israel O., Amiga E. (2014). Analysis of exhaled breath for diagnosing head and neck squamous cell carcinoma: A feasibility study. Br. J. Cancer.

[151] Bachar N., Liberman L., Muallem F., Feng X., Mullen K., Haick H. (2013). Sensor Arrays Based on Polycyclic Aromatic Hydrocarbons: Chemiresistors versus Quartz-Crystal Microbalance. ACS Appl. Mater. Interfaces.

[152] Fu X.A., Li M., Knipp R.J., Nantz M.H., Bousamra M. (2014). Noninvasive detection of lung cancer using exhaled breath. Cancer Med..

[153] Gouma P.I., Kalyanasundaram K. (2008). A selective nanosensing probe for nitric oxide. Appl. Phys. Lett..

[154] Nasution T.I., Nainggolan I., Hutagalung S.D., Ahmad K.R., Ahmad Z.A. (2013). The sensing mechanism and detection of low concentration acetone using chitosan-based sensors. Sens. Actuators B Chem..

[155] Liu C., Hayashi K., Toko K. (2012). Au nanoparticles decorated polyaniline nanofiber sensor for detecting volatile sulfur compounds in expired breath. Sens. Actuators B Chem..

[156] Neri G., Bonavita A., Micali G., Donato N. (2010). Design and development of a breath acetone MOS sensor for ketogenic diets control. Sens. J. IEEE.

[157] Pantalei S., Zampetti E., Bearzotti A., de Cesare F., Macagnano A. (2013). Improving sensing features of a nanocomposite PEDOT: PSS sensor for NO breath monitoring. Sens. Actuators B Chem..

[158] Toda K., Li J., Dasgupta P.K. (2006). Measurement of ammonia in human breath with a liquid-film conductivity sensor. Anal. Chem..

[159] Liu H.C., Huang W.C., Chen Y.J., Lu C.C., Huang J.T. (2013). An Electromechanical System Based on Carbon Nanotube Sensors to Detect Apnea. IEEE Sens. J..

[160] Kuzmych O., Allen B.L., Star A. (2007). Carbon nanotube sensors for exhaled breath components. Nanotechnology.

[161] Choi S.-J., Lee I., Jang B.-H., Youn D.-Y., Ryu W.-H., Park C.O., Kim I.-D. (2012). Selective Diagnosis of Diabetes Using Pt-Functionalized WO_3_ Hemitube Networks As a Sensing Layer of Acetone in Exhaled Breath. Anal. Chem..

